# A Manual of Qualitative Analysis

**Published:** 1872-09

**Authors:** 


*A Manual of Qualitative Analysis.* By ROBERT GALLOWAY, F. C. S. From the Fifth Re-written London Edition, with Illustrations. Philadelphia: HENRY C. LEA, 1872.

This work has been so long before the public and has been so favorably received and mentioned that it seems needless for us to give it anything of an extended notice.

The work is eminently practical in character, and has fully accomplished the author's object to furnish a guide for those commencing the study. It has in some respects accomplished more than this, and may be consulted with advantage by more advanced students.

This work is constructed on a plan to make the student "compare, distinguish, judge; so that thus, instead of being a recipient, he may become an intelligent and active discoverer."

